# Optimal Base Station Placement for Wireless Sensor Networks with Successive Interference Cancellation

**DOI:** 10.3390/s150101676

**Published:** 2015-01-14

**Authors:** Lei Shi, Jianjun Zhang, Yi Shi, Xu Ding, Zhenchun Wei

**Affiliations:** 1 School of Computer and Information, Hefei University of Technology, Hefei 230009, China; E-Mails: shilei@hfut.edu.cn (L.S.); dingxu@ialab.hfut.edu.cn (X.D.); wzc@ialab.hfut.edu.cn (Z.W.); 2 Intelligent Automation Inc., 15400 Calhoun Drive, Rockville, MD 20855, USA; E-Mail: yshi@vt.edu

**Keywords:** base station placement, wireless sensor network, interference management, successive interference cancellation

## Abstract

We consider the base station placement problem for wireless sensor networks with successive interference cancellation (SIC) to improve throughput. We build a mathematical model for SIC. Although this model cannot be solved directly, it enables us to identify a necessary condition for SIC on distances from sensor nodes to the base station. Based on this relationship, we propose to divide the feasible region of the base station into small pieces and choose a point within each piece for base station placement. The point with the largest throughput is identified as the solution. The complexity of this algorithm is polynomial. Simulation results show that this algorithm can achieve about 25% improvement compared with the case that the base station is placed at the center of the network coverage area when using SIC.

## 1. Introduction

Due to limited resources, it is a challenge to increase throughput in a wireless sensor network (WSN) [1]. Traditional interference avoidance mechanisms, e.g., TDMA, CDMA, CSMA, do not allow concurrent transmissions and, thus, cannot maximize throughput. Recently, interference management [2,3] was proposed to enable concurrent transmissions. Several techniques [4,5] were designed to realize such interference management. Among them, successive interference cancellation (SIC) [6] is easy to implement while also being able to achieve good performance. Zhang and Haenggi proposed a unified framework to study the performance of SIC [7]. Their analytical results showed that SIC can achieve potential gain in wireless networks, especially in heterogeneous cellular networks. Blomer and Jindal showed that although the benefit of SIC may be small for a small network with two links [8], the benefit is much larger for a complex network with many links [9]. Since SIC changes physical layer behaviors, new schemes and protocols should be designed for the upper layers, so that SIC can be used effectively [10]. For example, Lv *et al*. focused on link scheduling in *ad hoc* networks with SIC and developed a greedy scheduling algorithm, which can improve the throughput gain by 30% to 60% in simulations [11]. They later designed another algorithm [12] based on the simultaneity graph model and achieved a throughput gain from 40% to 120%. Jiang *et al.* proposed an optimal cross-layer algorithm that can be used in a multi-hop network with SIC and showed that their algorithm can increase throughput by 47% compared with the interference avoidance model [13]. Shi *et al*. proposed a greedy cross-layer algorithm for both scheduling and routing with polynomial complexity [14], and thus, it can be used for a multi-hop network with many nodes.

In addition to new upper layer schemes, base station location also has a significant impact on throughput, since data from sensor nodes will eventually transmit to the base station. The base station placement problems have also been studied by many researchers. Akkaya *et al*. studied positioning of base stations in WSNs [15]. They gave a survey of different base-station positioning works, categorized the literature into different approaches and then introduced dynamic schemes to repositioning base stations during the network operation. Arkin *et al*. proposed a heuristic for data transmission and base station placement to optimize network lifetime [16]. They built the model with a set of sensors and one base station, then studied the situation with at most two hops in the network and then extended it to multiple hops. Shi *et al*. designed a (1−*ε*)-optimal approximation algorithm [17] to improve the network lifetime for a multi-hop sensor network, where *ε* can be set as a very small number. They then considered that the base station can move in the network for collecting data and proposed another (1 − *ε*)-optimal approximation algorithm [18] for the base station moving path and data routing. To investigate a joint femtocell base station placement and power control problem, Liu *et al*. built a mixed-integer linear program (MILP) model for the femtocell network in commercial building environments [19]. They then proposed an optimization algorithm based on this model and showed that their algorithm had low complexity in simulation. Other researchers focused their interests on the base station problems in cellular networks and mainly on how to design energy-saving algorithms for base stations [20–22].

However, almost all existing works focused on either SIC or base station placement only, although we may further improve throughput by considering joint SIC and base station placement for a WSN. Zhou and Yu considered the influence of SIC on the base station in cellular networks [23] and proposed a scheme that performs Wyner–Ziv compress-and-forward relaying on a per base station basis. However, their work was on the uplink multi-cell joint processing technique, without consideration of choosing suitable positions for base stations. Altman *et al*. considered base station placement with SIC [24]. However, their work was mainly on the determination of base stations for mobile users in a cellular network. They only considered the model of hierarchical equilibria for SIC in two-frequency systems and do not consider the scheduling and routing method of the whole network after the determination of the base stations.

In this paper, we will design a base station placement algorithm for WSNs with SIC. We first formulate the problem and point out that the problem has prohibitive complexity, which makes it difficult to solve directly. Thus, we consider the relationship for distances between nodes and the base station under SIC. Based on this relationship, we propose an algorithm, which we called the likelihood maximum inscribed disk (LMID) algorithm, to find a good position for base station. The analysis for complexity shows that the LMID algorithm is polynomial-time, and simulation results show that the LMID algorithm can achieve about 25% improvement compared with our previous work [25] on SIC only.

The rest of the paper is organized as follows. In Section 2, we propose the mathematical model for the problem and find the relationship for distances between nodes and the base station under SIC. Section 3 presents the main idea and the details of the LMID algorithm. We also analyze its polynomial-time complexity in this section. In Section 4, we present simulation results to show the efficiency of our algorithm. Section 5 concludes this paper.

## 2. The Problem Model

We consider a WSN with *n* sensor nodes and one base station *B* in a two-dimensional area. Denote *N* as the set of sensor nodes. Sensor nodes are already deployed. Denote (*x_i_, y*_i_) as the location of each sensor node *si* ∈ *N*, which is fixed and known. Denote (*x_b_*, *y_b_*) as the location of the base station *B*, which can be optimized to improve sensor network performance. All nodes use the same power *P* and the same bandwidth *W* to transmit their data to *B* directly. Assume that each sensor node *s_i_* has a minimum rate requirement *r_i_*. We want to maximize a common scaling factor *K*, such that *s_i_* can transmit data to *B* with rate *Kr_i_*.

Denote $d_{i}=\sqrt{{\left(x_{i}-x_{B}\right)}^{2}+{\left(y_{i}-y_{B}\right)}^{2}}$ as the distance between node *s_i_* and *B, g_i_* as the channel gain from node *s_i_* to *B* and λ as the path loss index. We have:

(1) $$g_{i}=d_{i}^{-\lambda }$$

We will use the SIC technique [6] to improve throughput. By using SIC, multiple nodes may transmit at the same time to *B*, and their data will be decoded sequentially, based on their signal strength (from the strongest signal to the weakest signal). When we try to decode data from node *s_i_*, the stronger signals than *s_i_*'s must have already been decoded and removed via interference cancellation. Note that even with SIC, base station *B* may not be able to receive from all sensor nodes at the same time. Thus, we need to schedule sensor nodes in multiple (say *m*) time slots. The length of each time slot is *t*_1_, *t*_2_,…, *t_m_*, and the total length of time slots is *T, i.e*., $T=\sum_{i=1}^{m}t_{i}$. We now consider an SIC set *k*, where 1 ≤ *k* ≤ *m*, and define variable:

$$\theta_{i}^{k}=\{\begin{array}{ll} 1: & \text{if node}\;s_{i}\;\text{trasmits data to}\;B\;\text{in time slot}\;t_{k};\\ 0: & \text{otherwise}. \end{array}$$

Node *s_i_*'s signal-to-noise ratio (SINR) under SIC (say *σ_i_*) can be written as:

(2) $$\sigma_{i}^{k}=\frac{g_{i}\cdot \theta_{i}^{k}P}{N_{0}+\underset{g_{j}\leq g_{i}}{\text{∑}}g_{j}\cdot \theta_{j}^{k}P}$$

where *N*_0_ is the noise power. Note that there is interference from a node *s_j_* only if *g_j_* ≤ *g_i_*, since all stronger signals are decoded and removed under SIC. This SINR should be larger than a threshold (say *β*), such that it can be decoded with a peak data rate $W\log_{2}\left(1+\sigma_{i}^{k}\right)$.

We can normalize all *t_k_* by *T* and then have $\sum_{k=1}^{m}t_{k}=1$. For each node, its average data rate over all time slots should be no more than $\sum_{k=1}^{m}t_{k}W\log_{2}\left(1+\sigma_{i}^{k}\right)$. Then, we have the following problem.

(3) s.t.{gi=[(xi−xB)2+(yi−yB)2]−λ(si∈N)σik=gi⋅θikPN0+∑gj≤gigj⋅θjkP(si∈N,1≤k≤m)σik≥β(si∈N,1≤k≤m)Kri≤∑k=1mtkWlog2(1+σik)(si∈N)∑k=1mtk=1(xB,yB)∈A,gi,σik,tk,K≥0,θik∈{0,1}(si∈N,1≤k≤m)maxK

where *A* is an area of possible base station locations and will be determined in Section 3. *x_B_, y_B_, g_i_*, $\sigma_{i}^{k}$, *t_k_*, $\theta_{i}^{k}$ and *K* are all variables. This formulated problem is a mixed integer, non-convex program, which cannot be solved efficiently

In Equation (3), variables *g_i_* are calculated by a non-convex function. As a result, the optimization problem is non-convex. If we can determine the base station *B*'s position, then all of the *g_i_* values will become constants.

Furthermore, we can determine whether some nodes can transmit together or not by Equation (2). We have the following result on concurrent transmitting nodes [25].

### Theorem 1

*If multiple nodes can transmit at the same time and we sort them by their distances to B, then for two nodes s_i_ and s_j_ adjacent in the sorted list with d_j_* > *d_i_, we have* $\frac{d_{j}}{d_{i}}>\beta^{\frac{1}{\lambda }}$.

**Figure 1.**
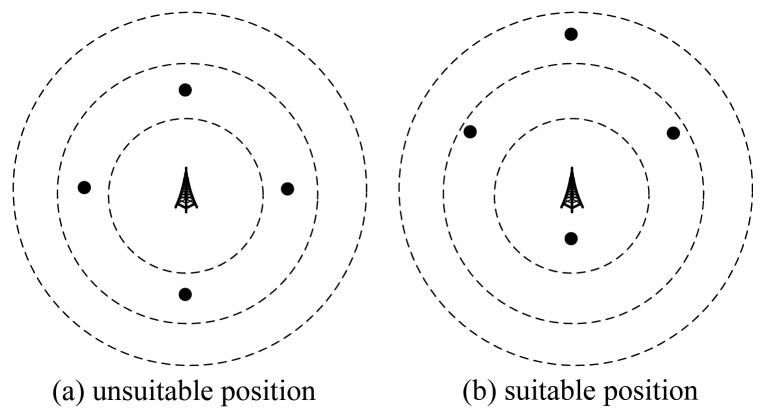
Suitable and unsuitable positions for the base station.

Based on Theorem 1, we can draw concentric disks around the base station. Nodes in the same concentric ring cannot transmit simultaneously. For example, consider a WSN with four nodes, as shown in Figure 1. In Figure 1a, the position of *B* is not a good position, because all nodes are in the same concentric ring, so these nodes cannot transmit together. While in Figure 1b, three of them can transmit together. Therefore, the position of *B* in Figure 1b is better than that in Figure 1a. Based on this example, we will design an algorithm in the next section.

## 3. The Base Station Placement Algorithm

Our algorithm includes two steps. First, we will determine the feasible region where we can place the base station, such that all sensor nodes can transmit data to the base station. Second, we will find a suitable position within the feasible region for the base station with the objective of maximizing the common scaling factor *K*. In this section, we will describe these two steps and analyze their complexities.

### 3.1. Find the Feasible Region for Base Station Placement

**Figure 2.**
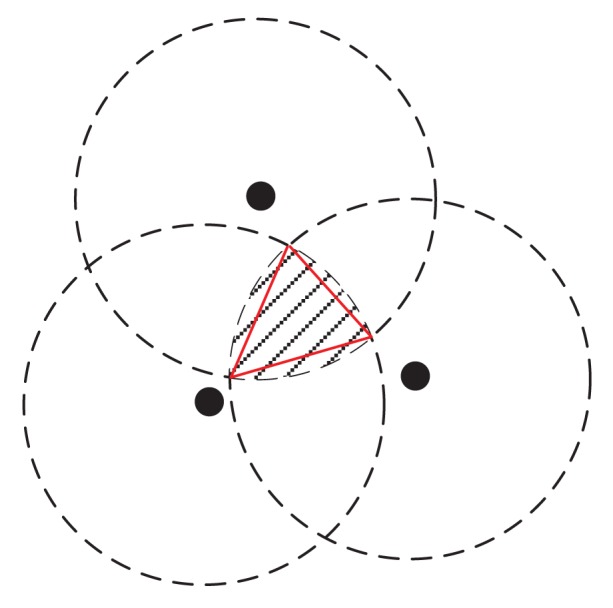
An example for the feasible region.

In a two-dimensional area, the base station cannot be placed arbitrarily, because there is a transmission range for all nodes. We have:

$$\frac{g_{i}P}{N_{0}}=\frac{d_{i}^{-\lambda }P}{N_{0}}\geq \sigma_{i}\geq \beta$$

where $\frac{d_{i}^{-\lambda }P}{N_{0}}=\sigma_{i}$ if there is no interference. For this ideal case, the transmission range *R_T_* can be calculated by $\frac{R_{T}^{-\lambda }P}{N_{0}}=\sigma_{i}=\beta$, *i.e.*, $R_{T}={\left(\frac{P}{N_{0}\beta }\right)}^{1/\lambda }$. Apparently, if the base station is out of the transmission range of a node, then this node's data cannot be transmitted to *B*. Thus, the base station *B* should be placed in a disk centered at this node with radius *R_T_*. Then, the feasible region of *B* is the intersection of all of these disks. One example is shown in Figure 2 for three nodes, where the shaded region is the feasible region for *B*. The boundary of this region is a set of arcs.

**Figure 3.**
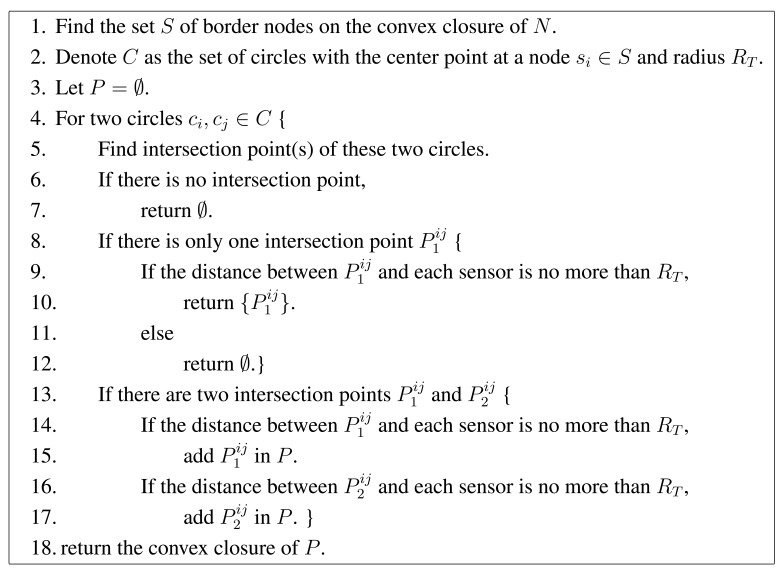
The pseudocode for finding the feasible region for *B*.

Instead of identifying the exact feasible region (or the set of arcs for this region), we can approximate it by a polygon, where each arc is approximated by a segment (see the triangle in Figure 2 as an example). The vertices of this polygon can be determined by the algorithm in Figure 3. This algorithm is based on the following two facts.

(1) To find the feasible region, we only need to find the convex closure of all nodes in *N* and consider the nodes on the border of the convex closure.
(2) The intersection points of two circles are vertices of the polygon if and only if they are within all disks.

To prove the first fact, we denote *p* as a feasible location for *B, S* as the set of nodes on the border of the convex closure of *N, s_i_* as a node in the network and *s_i_* ∉ *S.* The distance from *s_i_* to *p* is *d_i_*. Then, we can make a line from *p* to *s_i_*, and the line will intersect at a point *ŝ_i_* with *S*, where *d_i_* < *d_i_* ≤ *R_T_* . Thus, we do not need to consider the nodes that are not on the border of the convex closure. The convex closure can be determined by efficient algorithms, such as the algorithm named “Graham scan” [26].

There are two special situations for the second fact. First, any two circles have no intersection points. That means there is no feasible position for *B*. Second, any two circles have only one intersection point. Then, if the point is within all other disks, this point is the only feasible position for *B*. Otherwise, there is no feasible position for *B*.

**Figure 4.**
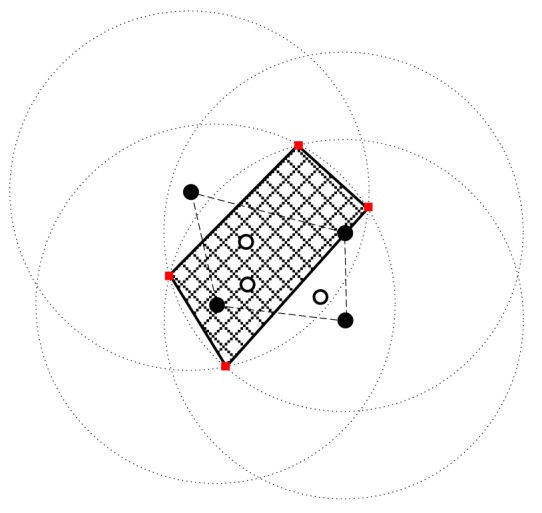
An example for finding a feasible region of *B*.

Figure 4 is a simple example for the algorithm in Figure 3. There are seven nodes in the network. Among them, the four black nodes are the vertices of the convex closure, while the three white nodes are inside the convex closure. Therefore, we only need to consider four black nodes to determine a feasible region. We obtain the four red nodes as the vertices of the feasible region.

### 3.2. The Main Idea for Finding a Good Position of B

We now determine a good position within the identified feasible region for *B*. As shown in Figure 1, a good position should enable more nodes to transmit together. We design a likelihood maximum inscribed disk (LMID) algorithm to determine *B*'s location. The main idea and the basic steps of the LMID algorithm are as follows.

I. The set of locations that have the same distance to two nodes is the perpendicular bisector of the line segment between these two nodes. We should not put the base station in (or close to) this perpendicular bisector. Otherwise, these two nodes cannot transmit at the same time. All such perpendicular bisectors divide the feasible region into many small pieces.
II. Find a point in each piece, such that it has the largest distances to all edges of this piece.
III. Put the base station at each point found in Step II and calculate all *K* values. Find the maximum value among these values and use the corresponding point as the solution for *B*'s position.

We first prove that each small piece obtained in Step I is a convex polygon. Note that the feasible region is a convex polygon (since it is a convex closure of some points). We can add perpendicular bisectors one by one to divide this feasible region. We have the following lemma.

#### Lemma 1

*After we use a perpendicular bisector to divide a convex polygon into two pieces, these two pieces are both convex polygons*.

##### Proof

**Figure 5.**
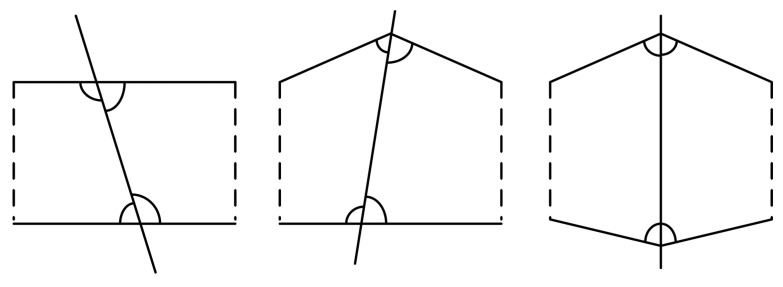
Three situations when dividing the region.

As a convex polygon, all inner angles are less than 180 degree. There are three different situations when a line (perpendicular bisector) divides a convex polygon into two pieces, as shown in Figure 5: (I) the line intersects with two edges of the convex polygon; (II) the line intersects with one edge and one angle of the convex polygon; (III) the line intersects with two angles of the convex polygon. No matter the situation, all new angles of the two new pieces are less than 180 degree. Thus, the two pieces are both convex polygons.

To find the point in Step II, we have the following algorithm.

**Figure 6.**
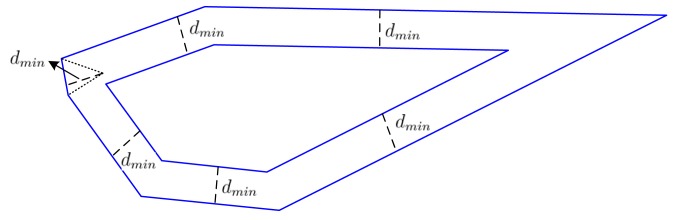
A example of shrinking.

A. The bisectors of two adjacent interior angles intersect at one point. Calculate the distance *d* from this point to the common edge of the two angles.
B. We have one distance for each edge in Step A. Find the minimum distance *d_min_* among these distances. Delete this edge by coalescing its two endpoints into one point located at the common point of bisection. Then, shrink the polygon by moving all of the remaining edges into the polygon's interior by *d_min_*. Since the edge corresponding to *d_min_* is reduced to a point, the number of edges of the new polygon is reduced by at least one. Figure 6 is an example of a six-edge polygon shrinking into a five-edge polygon.
C. If the new polygon has more than three edges, go to Step A. If the new polygon has three edges, it is a triangle. We find the center point of the maximum inscribed disk for the triangle as the base station location. If the new polygon reduces as a line segment, we choose any point on this segment as the base station location. If the new polygon reduces as a point, this point is chosen as the base station location.

If *n* = 3, the center point of the maximum inscribed circle for the triangle has the largest distances to all edges, and thus, it is a good position. For an n-edge polygon, *n* > 3, we also want to identify its maximum inscribed disk. We have the following theorem.

#### Theorem 2

*Suppose that we reduce an m-edge convex polygon to an k-edge convex polygon, k* < *m, by Steps A and B, and we know the maximum inside disk of the k-edge polygon. Then, the center point of this disk is also the center point of a maximum inside disk for the m-edge polygon*.

##### Proof

The proof is based on contradiction. Suppose that the maximum inside disk for the k-edge polygon has the center point *O_k_* and radius *r_k_*. Denote the shrink distance for the *m*-edge polygon as *d_m_*. Then, we have an inside disk for the *m*-edge polygon with center *O_k_* and radius *r_k_* + *d_m_*. Suppose that the center point of the maximum inside disk for the *k*-edge polygon is not the center point of a maximum inside disk for the *m*-edge polygon. That is, the maximum inside disk for the *m*-edge polygon has radius *r_m_* larger than *r_k_* + *d_m_*. Now, reduce the radius of this maximum inside disk for the *m*-edge polygon by d_m_; we have an inside disk for the *k*-edge polygon with radius *r_m_* − *d_m_* > *r_k_*. This is a contradiction.

Step II provides many potential good points for the base station. In Step III, we will calculate the *k* value for each point based on the algorithm developed in [25] and then find the best point.

### 3.3. Complexity

We will first analyze the complexity for finding the feasible region (see the pseudocode in Figure 3). First, we need to find the convex closure of nodes in set *N* in Step 1, which can be done by many algorithms. In particular, the complexity of the “Graham scan” algorithm is *O*(*n*^2^ log *n*^2^) = *O*(*n*^2^ log *n*). The worst case is *S* = *N*. Under this worst case, the number of iterations (from Step 5 to Step 17) is *O*(*n*^2^). Within an iteration, the complexity to calculate the intersection points of two circles (Step 5) is *O*(1). If there is one intersection point, we need to check the distance from this point to each sensor (Step 9). The complexity is *O*(*n*). If there are two intersection points, we need to check the distance from these points to each sensor (Steps 14 and 16). The complexity is again *O*(*n*). Then, the total complexity of one iteration is *O*(*n*), and the complexity of all iterations is *O*(*n*)*O*(*n*^2^) = *O*(*n*^3^). Finally, we need to find the convex closure of the nodes in set *P*. If we use the “Graham scan” algorithm, the complexity is *O*(*p*^2^ log *p*^2^) = *O*(*p*^2^ log *p*) = *O*(*n*^4^ log *n*), where *p* is the number of nodes in set *P* and is no more than 2*O*(*n*^2^) = *O*(*n*^2^). The overall complexity is *O*(*n*^2^ log *n*) + *O*(*n*^2^)*O*(*n*^3^) + *O*(*n*^4^ log *n*) = *O*(*n*^5^).

Next, we will show the complexity for finding the suitable positions by LMID.

For the first step, the number of the perpendicular bisectors is no more than *O*(*n*^2^), where *n* is the number of nodes in the network. Using these perpendicular bisectors to divide the feasible region, the number of the pieces is no more than:

$$\frac{O\left(n^{2}\right)\left(O\left(n^{2}\right)+1\right)}{2}+1=O\left(n^{4}\right)$$

The second step is further divided into three substeps. For Step (A), the number of different distances is *O*(*n*). For Step (B), *d_min_* can be determined in *O*(*n*). Then, the complexity of shrink is also *O*(*n*). Since at least one edge is removed by one iteration of Steps (A) and (B), we will repeat Steps (A) and (B) *O*(*n*) times. The complexity of Step (C) is *O*(1). Then, the overall complexity of the second step is (*O*(*n*) + *O*(*n*))*O*(*n*) + *O*(1) = *O*(*n*^2^).

Combining the first and the second steps, the complexity of finding all possible positions is *O*(*n*^4^) × *O*(*n*^2^) = *O*(*n*^6^). Therefore, the whole complexity of our algorithm is *O*(*n*^5^) + *O*(*n*^6^) = *O*(*n*^6^), which is polynomial. This result is a worst-case upper bound.

## 4. Simulation Results

In this section, we show simulation results on the performance of our algorithm. We also compare results with our previous work in [25], where the location of the base station is fixed at the center of the network coverage area. We first present one set of results on a network with 20 nodes, then give all sets of results on networks with 10 to 50 nodes.

We randomly generate network instances with nodes deployed in a square region of 250 × 250 m. Transmission power is *P* = 1 W; the path loss index is λ = 4; noise power is *N*_0_ = 10^−10^ W; and the SINR threshold is *β* = 3. The transmission range of each node *r_t_* can be calculated as $R_{T}={\left(\frac{P}{N_{0}\beta }\right)}^{1/\lambda }\approx 240\text{m}$. The channel bandwidth is *W* = 22 MHz. The required minimum data rate *r_i_* is randomly generated between 100 kbps and 1 Mbps.

### 4.1. Results for a Network with 20 Nodes

**Figure 7.**
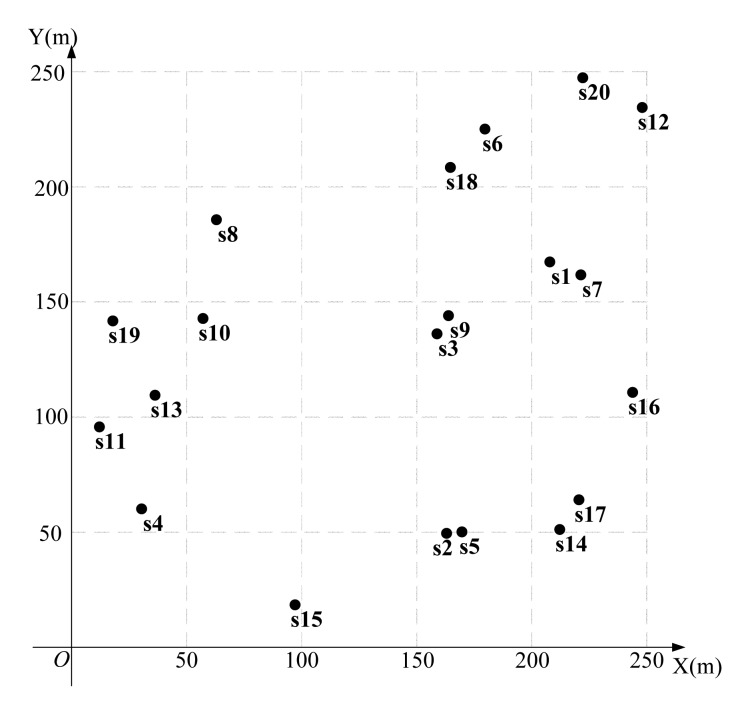
A 20-node network.

We first present results for a network with 20 nodes, shown in Figure 7. The coordinates and the required minimum data rate *r_i_* are randomly generated and shown in Table 1.

**Figure 8.**
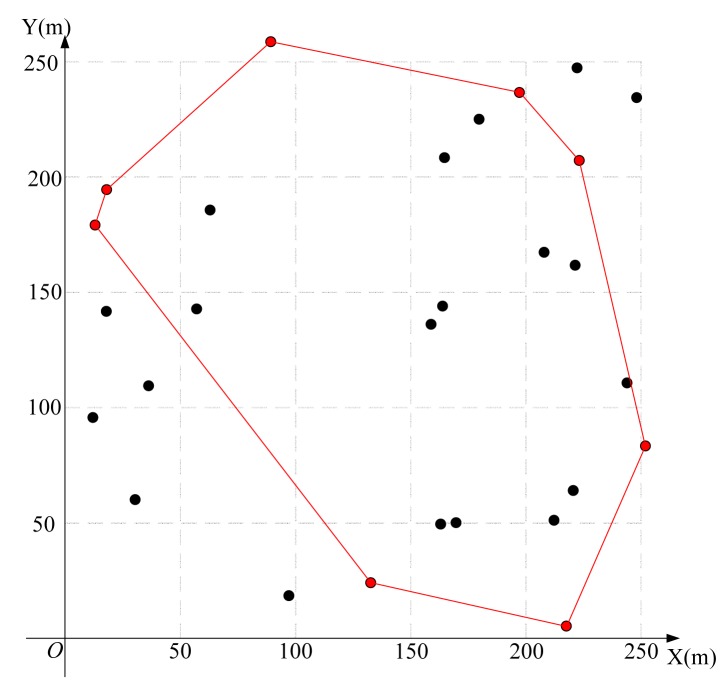
Feasible region for the 20-node network.

**Figure 9.**
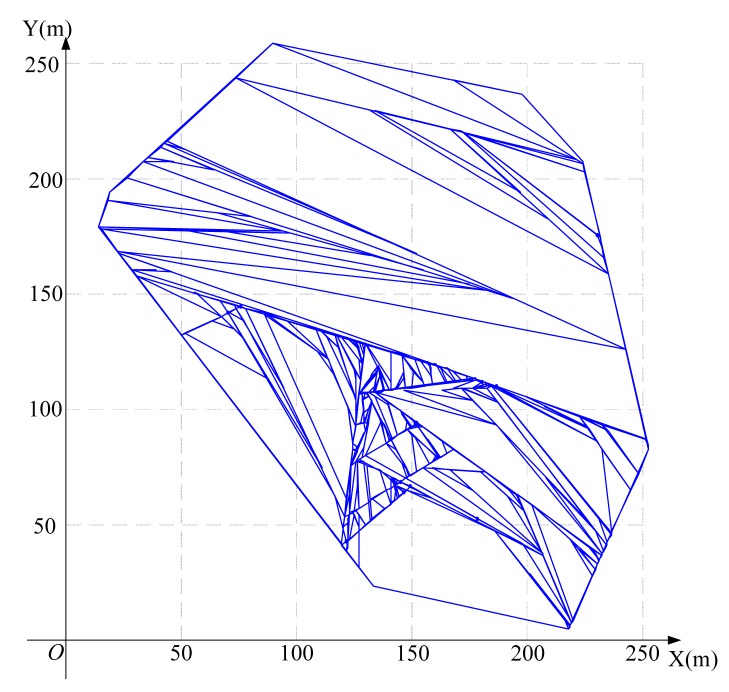
Dividing the feasible region into pieces for the 20-node network.

**Figure 10.**
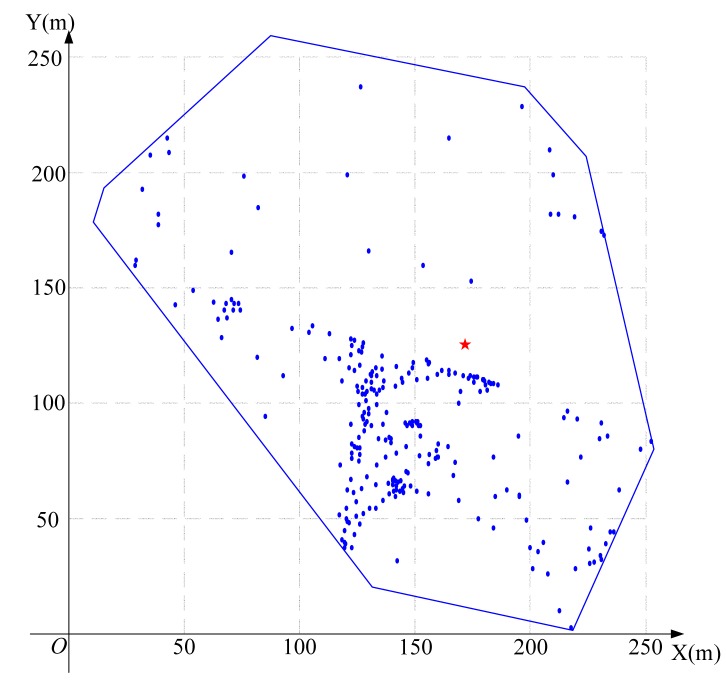
Center points of all pieces for the 20-node network.

Using the algorithm in Figure 3 to find the feasible region for base station placement, the vertex coordinates of the feasible region are (223.3,207.4), (197.4,236.3), (252.1,83.7), (14.4,179.6), (133.0,25.0), (89.6,258.2) (19.1,193.9) and (217.7,6.8), as shown in Figure 8. Then, using the LMID algorithm, we divide the feasible region into 262 pieces, as shown in Figure 9. The center points of these pieces are shown in Figure 10. We then calculate the *K* value for the case that the base station is placed at each of these center points. We find that the maximum value of *K* is 23.151 when the coordinate of *B* is (171.9,127.8). We compare the performance with and without base station placement, where the second case can be solved by the algorithm in [25] and the base station is fixed at the center of the region (*i.e*., (125,125)). When the base station is placed at the center of the region, the *K* value is 16.592 with SIC and 4.119 without SIC. They are both less than that with base station placement.

**Figure 11.**
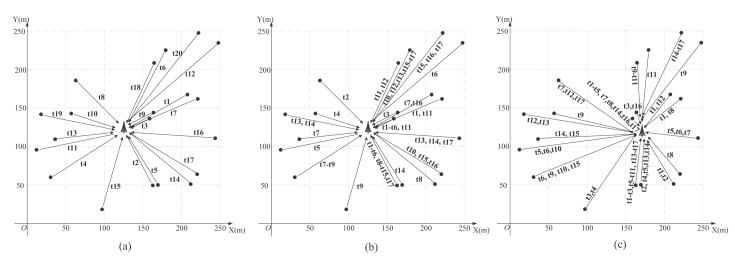
Link scheduling for the 20-node network with the three schemes.

Figure 11 shows the link scheduling for the 20-node network with the three schemes. Figure 11a is for the scheduling without SIC when the base station is in the center of the network. Figure 11b is for the scheduling with SIC when the base station is in the center of the network, and Figure 11c is for the scheduling with SIC when using the LMID algorithm. We show the time slot numbers on each link. From this, we can see in Figure 11a that each node is assigned a different time slot for transmission, so the total number of time slots is 20; while in Figure 11b,c, some nodes can transmit at the same time slot, and the total numbers of the time slots are both 17. We record the length of each time slot in Table 2.

### 4.2. Results for All Network Instances

To show the performance of our algorithm, we change the number of nodes *N* from 10 to 50 and generate 20 different network instances randomly for each *N*. Then, we use the LMID algorithm to find a good position of *B* for each network instance and calculate the achieved *K* value. We calculate the average values for each *N* in Table 3, where the second column is for the scheme when the base station is fixed and there is no SIC, the third column is for the scheme when the base station is fixed and with SIC and the fourth column is for the scheme when using the LMID algorithm. We can see that the LMID algorithm has much better performance (the value of K) than the fixed base station without SIC. Even if we compare LMID with the fixed base station with SIC, LMID can still achieve about a 25% improvement.

## 5. Conclusions

In our previous work, we applied the SIC technique for interference management and proposed algorithms of link scheduling and data routing schemes for different models with fixed base stations [14,25]. In this paper, we considered base station placement in wireless sensor networks. Since the mathematical model using SIC was non-convex and could not be solved directly, we proposed an algorithm, which we call the likelihood maximum inscribed disk (LMID) algorithm, to solve it. The LMID algorithm is based on the relationship of distances from sensor nodes to the base station under the SIC environment, and we show that it can find a good base station position in polynomial time. Simulation results show that the LMID algorithm can increase throughput by about 25% compared with the case that the base station is located at the center of the region.

## Figures and Tables

**Table 1.** Coordinates (m) and *r_i_* values (kbps) of all nodes in the 20-node network.

*i*	**Coordinates**	*r_i_*	*i*	**Coordinates**	*r_i_*
1	(207,172)	666.369	11	(12,93)	845.894
2	(164,50)	386.032	12	(248,236)	729.633
3	(159,139)	350.260	13	(36,110)	322.546
4	(32,62)	221.291	14	(213,52)	405.357
5	(171,51)	229.944	15	(97,18)	775.751
6	(181,228)	532.300	16	(245,112)	444.750
7	(224,163)	703.341	17	(221,66)	517.417
8	(64,189)	746.221	18	(167,210)	639.194
9	(166,142)	389.072	19	(19,142)	845.549
10	(59,141)	128.535	20	(225,247)	607.315

**Table 2.** The length of each time slot for the three schemes.

*i*	**Length for (a)**	**Length for (b)**	**Length for (c)**	*i*	**Length for (a)**	**Length for (b)**	**Length for (c)**
1	0.081	0.031	0.056	11	0.023	0.010	0.060
2	0.020	0.072	0.068	12	0.173	0.050	0.044
3	0.025	0.069	0.151	13	0.010	0.015	0.012
4	0.005	0.084	0.0002	14	0.031	0.096	0.049
5	0.012	0.102	0.034	15	0.022	0.035	0.096
6	0.079	0.096	0.042	16	0.054	0.030	0.059
7	0.092	0.028	0.087	17	0.044	0.078	0.024
8	0.049	0.065	0.070	18	0.077		
9	0.030	0.052	0.126	19	0.034		
10	0.006	0.086	0.024	20	0.134		

**Table 3.** Optimization results for base station placement.

*n*	**Center** *B* **without SIC**	**Center** *B* **with SIC**	**Placement with SIC**	**Improvement Comparing with Center** *B* **with SIC**
10	13.2	47.0	59.2	26.0%
15	7.9	30.4	37.9	24.5%
20	5.4	22.4	28.4	27.0%
25	4.9	19.8	25.0	26.2%
30	3.7	16.0	21.0	31.3%
35	3.0	13.1	16.7	27.4%
40	2.9	12.4	15.3	22.8%
45	2.6	11.5	14.1	23.2%
50	2.3	10.1	12.7	25.0%
